# Symptomatic Differences between Influenza A/H3N2 and A/H1N1 in Korea

**DOI:** 10.3390/jcm12175651

**Published:** 2023-08-30

**Authors:** Hyun-Jong Lee, Gwanghui Ryu, Ki-Il Lee

**Affiliations:** 1 Lee and Hong ENT, Sleep and Cosmetic Center, Seongnam 13558, Republic of Korea; henjoi.lee@gmail.com; 2 Department of Otorhinolaryngology-Head and Neck Surgery, Samsung Medical Center, Sungkyunkwan University School of Medicine, Seoul 06351, Republic of Korea; ghryu379@gmail.com; 3 Myunggok Medical Research Institute, Konyang University College of Medicine, Daejeon 35365, Republic of Korea; 4 Department of Otorhinolaryngology-Head and Neck Surgery, Konyang University College of Medicine, Daejeon 35365, Republic of Korea

**Keywords:** influenza A, virus, subtype, primary health care, rapid diagnostic tests

## Abstract

Background: Limited understanding exists regarding clinical distinctions between influenza A/H3N2 and A/H1N1 subtypes, particularly in primary health care. We conducted a comparative analysis of symptomatic characteristics of influenza subtypes in Korea. This retrospective study analyzed medical records of patients who presented with positive test results for influenza-like illness (rapid influenza diagnostic test; RIDT) during the H3N2-dominant 2016–2017 and H1N1-dominant 2018–2019 seasons. Symptomatic manifestations, contact history, vaccination history, and clinical course were analyzed between the two seasons. The most frequent symptom in the RIDT-positive patients was fever (80.1% and 79.1%, respectively). The average body temperature was higher, and the number of patients with high fever was greater in the H3N2-dominant season than in the H1N1-dominant season (*p* < 0.001). Conversely, other symptoms, such as myalgia, cough, and sore throat, were significantly more common in the H1N1-dominant season than in the H3N2-dominant season (*p* < 0.001). Antiviral drugs were prescribed to most febrile RIDT-positive patients (82.2% and 81.3%, respectively, *p* = 0.516). Analyzing primary care data revealed different clinical manifestations according to the subtype. Therefore, physicians should consider these variable hallmarks and employ tailored therapeutic strategies to reduce the complication rate.

## 1. Introduction

Influenza is prevalent worldwide and can lead to substantial morbidity and mortality. Individuals across all age groups are susceptible throughout their lives, with older individuals experiencing more severe complications [1,2]. Influenza is usually a self-limiting illness; however, influenza virus can undergo mutations and evolve continuously. Throughout history, certain variations, such as the “Spanish flu” in 1918, have evolved into pandemic outbreaks with fatal consequences [3]. The H1N1 subtype also led to a pandemic in 2009. Fortunately, unlike many other respiratory viruses, specific treatment options, such as a neuraminidase inhibitor (oseltamivir) and vaccinations, exist.

Environmental factors, including season or location, are important for the treatment and prevention of influenza. Different regions exhibit varying patterns of the illness. For instance, H1N1 infection tends to be relatively common and severe in Asia, whereas H3N2 infection is more prevalent and severe in Europe [4]. Furthermore, seasonality is associated with differing symptom profiles, even within the same area.

Although influenza usually follows a self-limiting course and resembles common colds induced by other respiratory viruses, most influenza studies have been conducted from a hospital-based or national healthcare perspective [5,6]. Conversely, influenza is a community-based illness commonly occurring in the winter. Consequently, for public health considerations, analyzing influenza at the primary care level is paramount. Currently, a deficit exists in well-designed studies concerning influenza at the primary care level.

The Korean Disease Control and Prevention Agency is enhancing its surveillance of influenza and other respiratory viruses. They are expanding their collection of specimens that cause respiratory symptoms. Based on the Korea Influenza and Respiratory Viruses Surveillance System, the initial detection week and epidemic subtype of the influenza virus were as follows: A/H3N2 in week 37 of the 2016–2017 season and A/H1N1 in week 36 of the 2018–2019 season [7].

The rapid influenza diagnostic test (RIDT) is a useful diagnostic tool for screening influenza in a clinical setting. It is important in primary care clinics to distinguish influenza from other upper respiratory infections. We conducted a multicenter study involving a primary care survey on influenza using the RIDT in a clinical setting. We aimed to compare the clinical characteristics of influenza according to different subtypes (H1N1 vs. H3N2) dominant across two different seasons.

## 2. Materials and Methods

### 2.1. Study Design

The survey was conducted by 15 otorhinolaryngologists in primary clinics in Seoul and Gyeonggi Provinces. They retrospectively reviewed medical records of patients who presented with influenza-like illness (febrile sense with respiratory symptoms) and tested positive on the RIDT. Two influenza outbreak seasons were included in this study, spanning from November to February of 2016–2017 and 2018–2019.

At each clinic, we compiled the following clinical data: age, sex, symptom onset, and clinical manifestations, including febrile sensation, body temperature, myalgia, cough, sore throat, and diarrhea. Moreover, we analyzed clinical information regarding history of contact with infected individuals, influenza vaccination history, oropharyngeal findings, antiviral medication (oral oseltamivir or intravenous peramivir) administration, and the clinical course following antiviral drug administration. Only patients with documented clinical courses involving factors such as fever subsidence, comorbidities, or complications were included in the study cohort. Details such as swabbing sites and methods, RIDT brands used, and test results (positive/weak, positive/negative) were also documented. Patients who were pregnant, had undergone sinonasal surgery, had underlying upper airway infection, or had other systemic infections were excluded from the study cohort. This study was approved by the Institutional Review Board (IRB No. 2020-09-016), and the need for informed consent was waived owing to the retrospective study design.

### 2.2. RIDTs

Upper airway samples were collected using nasopharyngeal or throat swabs. Various test kits were used in each clinic, including QuickNavi-Flu (Denka Seiken, Tokyo, Japan), GENEDIA Multi Influenza Ag rapid test (Green Cross Medical Science, Yongin, Republic of Korea), Humasis Influenza A/B antigen test (Humasis, Anyang, Republic of Korea), Biocredit influenza A and B (RapiGEN, Gunpo, Republic of Korea), BD Veritor System Flu A + B (BD Diagnostics, Sparks, MD, USA), SD Bioline rapid influenza kit (Standard Diagnostics, Yongin, Republic of Korea), JW easy test influenza A/B (JW Pharmaceutical, Seoul, Republic of Korea), Capilia Flu A + B (Taunus Laboratories, Shizuoka, Japan), ImmunoAce Flu (Tauns Laboratories), Alsonic Flu (Al- fresa Pharma Co., Osaka, Japan), SGT i-flex influenza A&B test (Sungentech Inc., Daejeon, Republic of Korea), and Quidel Sofia Influenza A + B FIA (Quidel Corp., San Diego, CA, USA). These RIDT kits can help distinguish between influenza A and B. The results, categorized as positive, weak positive, or negative, were typically available in approximately 10 min. For statistical analysis, a weak positive result was considered positive.

### 2.3. Diagnostic Methods for Comorbidity

All comorbidities were clinically diagnosed in this study. Upper airway comorbidities, including sinusitis, tonsillitis, otitis media, and laryngitis, were diagnosed by qualified otolaryngologists employing otolaryngic telescopes or microscopes for precise evaluation. Lymphadenitis was assessed through routine physical examination. However, in cases with suspected pneumonia, patients were referred to internal medicine physicians. These instances were subsequently confirmed based on physical examination with the aid of a stethoscope and chest radiography findings. This comprehensive and reliable diagnostic approach ensured the accurate identification of pneumonia. Moreover, patients exhibiting severe symptoms, such as general weakness or poor oral intake, were promptly referred to secondary or tertiary hospitals for specialized care. Subsequently, these patients received treatment through hospitalization, ensuring the comprehensive management of their condition in a more specialized healthcare setting.

### 2.4. Statistical Analyses

Continuous data are presented as means and standard deviations according to a normal distribution, as assessed using the Shapiro–Wilk test. Otherwise, data were presented as medians and interquartile ranges (IQRs). Continuous variables were analyzed using the Mann–Whitney U test. Categorical variables were analyzed using the chi-squared test or Fisher’s exact test. A *p*-value < 0.05 was considered statistically significant. Statistical analyses were performed using the STATA software v16.0 (StataCorp LP, College Station, TX, USA).

## 3. Results

### 3.1. Participants

Of a total of 4088 participants (1897 patients with influenza-like illness in the 2016–2017 season with H3N2 dominance and 2191 patients with influenza-like illness in the 2018–2019 season with H1N1 dominance), 1424 in the 2016–2017 season and 1568 in the 2018–2019 season with RIDT-positive findings were enrolled in this study. The demographic data are presented in Table 1. The participants were significantly younger in the 2016–2017 season compared with those in the 2018–2019 season (17 years vs. 27 years, respectively, *p* < 0.001). No sex difference was observed between the two seasons (*p* = 0.343). The history of contact with an infected person, such as family or friends, was recorded to be 4.7% in the 2016–2017 season and 8.1% in the 2018–2019 season (*p* < 0.001). The primary swab site was often the nasopharynx via the nose, with other swab sites including the oropharynx through the mouth or both the nasopharynx and oropharynx. The positive RIDT rate was slightly higher during the 2016–2017 season (71.8% vs. 65.0%, *p* < 0.001).

The percentage of participants who received an influenza vaccination was 23.9% in the 2016–2017 season (22.1% and 1.8% before and within 2 weeks, respectively) and 26.5% in the 2018–2019 season (26.2% and 0.3% before and within 2 weeks, respectively) (*p* < 0.001).

### 3.2. Initial Symptoms

**Figure 1:**
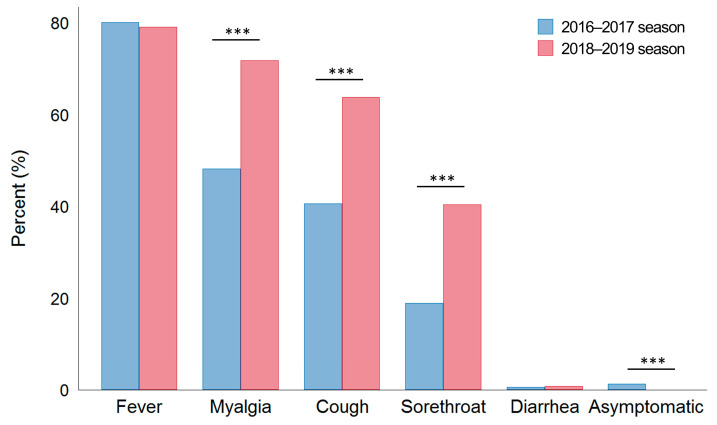
Initial manifestations according to subtype. Data are expressed as the means. *** *p* < 0.001 by the chi-squared test or Fisher’s exact test.

The most common symptom was fever in both seasons (80.1% vs. 79.1%, *p* = 0.496) (Figure 1 and Table 2). The average body temperature was higher in the 2016–2017 season than in the 2018–2019 season (38.1 °C vs. 37.9 °C, *p* < 0.001). The number of patients with temperatures > 38 °C was significantly higher in the 2016–2017 season than in the 2018–2019 season.

Interestingly, myalgia, cough, and sore throat were more frequently observed in the 2018–2019 season than in the 2016–2017 season (*p* < 0.001; Figure 1).

**Figure 2:**
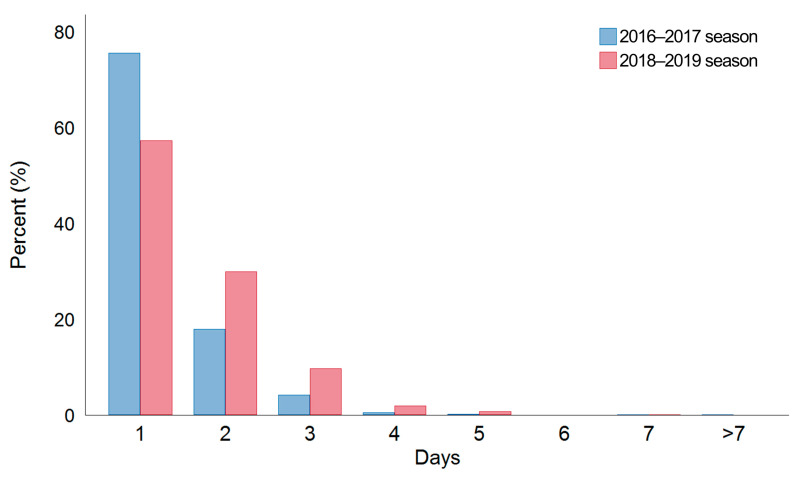
Average day of the first hospital visit after symptom onset. Data are expressed as the means.

In terms of hospital visits, most patients visited the clinic one or two days after symptom onset (88.9%) in both seasons (Figure 2). Hence, the average day of first hospital visit after symptom initiation was found to be 1.5 days (1.3 ± 0.7 (mean ± standard deviation) days in the 2016–2017 season and 1.6 ± 1.0 days in the 2018–2019 season).

### 3.3. Clinical Course

A total of 2446 patients (1171 patients in the 2016–2017 season and 1275 patients in the 2018–2019 season) were prescribed antiviral drugs (Table 3). No statistically significant difference was noted in the prescription rate of antiviral drugs between the two seasons (*p* = 0.516). Fever subsided within 24 h after antiviral treatment administration in 59.0% of patients in the 2016–2017 season and in 55.5% of patients post-24 h in the 2018–2019 season. Vomiting occurred in 26 (1.8%) and 31 (2.0%) patients after oseltamivir use in the 2016–2017 and 2018–2019 seasons, respectively.

**Figure 3:**
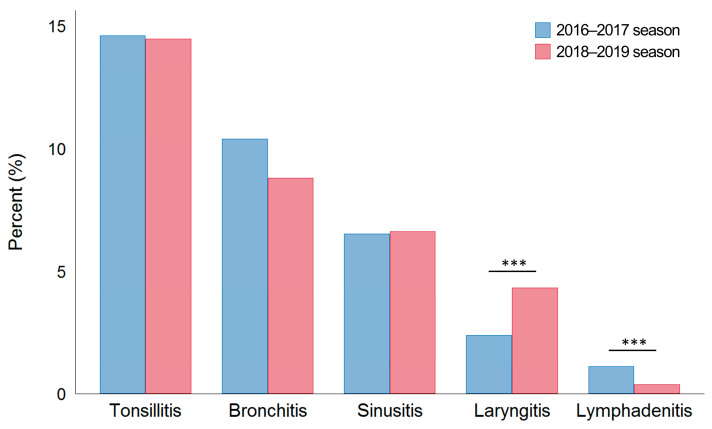
Upper airway comorbidities associated with influenza A by season. Data are expressed as means. *** *p* < 0.001 by the chi-squared test or Fisher’s exact test.

The prevalence of upper airway comorbidities, such as tonsillitis, bronchitis, sinusitis, laryngitis, and cervical lymphadenitis, was similar in the two seasons (tonsillitis, *p* = 0.092; bronchitis, *p* = 0.139; sinusitis, *p* = 0.911; and otitis media, *p* = 0.226) (Figure 3 and Table 4).

In the 2016–2017 season, tonsillitis was the most common (208 patients, 14.6%), followed by bronchitis (148 patients, 10.4%), sinusitis (93 patients, 6.5%), laryngitis (34 patients, 2.4%), and cervical lymphadenitis (16 patients, 1.1%) (Table 3 and Figure 3). Two patients had otitis media during the 2016–2017 season.

Similarly, in the 2018–2019 season, tonsillitis was the most frequent upper airway comorbidity (227 patients, 14.5%), followed by bronchitis (138 patients, 8.8%), sinusitis (104 patients, 6.6%), laryngitis (68 patients, 4.3%), and cervical lymphadenitis (six patients, 0.4%).

Furthermore, systemic complications, such as pneumonia, in one and three patients were observed in the 2016–2017 and 2018–2019 seasons, respectively. Hospitalization was necessary for one and five patients in the 2016–2017 and 2018–2019 seasons, respectively.

## 4. Discussion

Although there exist symptomatic hallmarks for respiratory virus infections, including influenza, physicians often opt for a conservative approach without considering individual virus subtypes. Nevertheless, the findings of this study suggest that physicians in Korea can leverage specific information about influenza subtypes from the national surveillance system to enhance their preparedness. Furthermore, they can provide accurate information regarding the epidemic subtype to their patients during clinical encounters.

Specifically, our study revealed that fever is the most prevalent symptom across both influenza A subtypes. Moreover, we observed a higher average body temperature during the A/H3N2 season compared with the A/H1N1 season. Additionally, myalgia, cough, and sore throat were relatively more common during the A/H1N1 season. However, no significant differences were detected in rates of sinusitis, tonsillitis, bronchitis, otitis media, pneumonia, and hospitalization between the two seasons.

Crucially, this study stands out as the first to compare influenza A subtypes (A/H3N2 vs. A/H1N1) at the primary clinic level, providing valuable insights for clinical management and decision making.

Previous research on influenza has documented varied manifestations depending on the healthcare setting (outpatient or emergency department or primary care/general hospital) [2,8]. Irving et al. [9] reported comparable clinical manifestations in outpatients with influenza A and B infections. Martinez et al. [10] asserted that older age and comorbidities correlated closely with severe outcomes based on an analysis of hospitalized patients with influenza. Similarly, in emergency departments, patients with influenza tend to be older and exhibit a higher prevalence of underlying diseases [11]. In the present study, we analyzed the clinical features of influenza diagnosed through RIDT and focused on primary otorhinolaryngology clinics.

In terms of subtype classification, influenza A virus can be categorized as having low (seasonal H3N2 and H1N1) or high virulence (1918 H1N1, H5N1, and likely 2009 H1N1) [12]. Pathologically, low-virulence viruses lead to primary inflammation, congestion, and epithelial necrosis of the larger airways, such as the trachea, bronchi, and bronchioles. Conversely, high-virulence viruses can provoke inflammation at the alveolar level. Among low-virulence viruses, experimental studies in pigs revealed that macroscopic and microscopic lesions produced by seasonal H1N1 were more extensive than those produced by H3N2 viruses [13].

From a clinical perspective, in contrast to pathology, patients with A/H3N2 tend to experience more severe disease than those with A/H1N1 [14]. However, influenza B has a higher incidence of gastrointestinal symptoms and may cause myalgia [15]. Yang et al. [16] reported no discernable symptomatic differences between outpatients and inpatients with seasonal and pandemic influenza A (A/H1N1) infections.

The level of social activity may be associated with the propagation of the virus within social networks and communities. Our study revealed a significant age disparity between patients in the A/H1N1-dominant season and those in the A/H3N2-dominant season. Specially, patients affected during A/H1N1-dominant seasons had a mean age in the mid-twenties, suggesting a socially active demographic. Conversely, patients impacted during the A/H3N2-dominant season were predominantly teenagers, indicating a relatively younger and potentially less socially active group. These findings suggest that diverse patterns of social activity within different age groups might contribute to the differential transmission of influenza strains. However, further investigation is necessary to establish a definite causal relationship and explore other potential factors influencing influenza prevalence among different demographics. This study underscores the significance of considering social dynamics and age-related variations in influenza surveillance and public health interventions.

In terms of hallmark symptoms, fever emerged as the most frequently reported symptom in this study, aligning with previous research [17]. Interestingly, the average body temperature and count of patients with high fever (>38 °C) were higher in the H3N2-dominant season than in the H1N1-dominant season. These findings are consistent with earlier studies suggesting that the H3N2 infection is more severe than the H1N1 infection, particularly fever [14]. Conversely, myalgia, cough, and sore throat were more commonly manifested in the H1N1-dominant season compared with the H3N2-dominant season. This observation might be attributed to the diverse nature of the A/H1N1 subtype among Asians [4].

Previous investigations have reported that the viral loads in the influenza A infection typically peak at approximately 1–2 days after symptom onset in adults [18,19]. Our study revealed that individuals sought medical attention sooner after symptom onset during the A/H3N2-dominant season compared with the A/H1N1-dominant season. Fever exhibited a lower threshold for tolerance compared with other respiratory manifestations, such as cough, sputum, or sore throat. Specifically, during the A/H3N2-dominant season, fever was more prevalent than any other symptom studied.

Recently, the RIDT has gained traction for influenza detection in clinical settings. Several studies using RIDT have reported outcomes comparable with those achieved with the polymerase chain reaction (PCR) method [20,21]. Clinical results using the RIDT were obtained within approximately 20 min, making it a valuable time-saving tool, especially in primary clinics where obtaining PCR results may take 2–3 days.

The nasopharynx stands out as the primary site for viral load in most patients. Although nasopharyngeal aspirate suctioning is superior for virus detection compared with nasal, nasopharyngeal, or oropharyngeal swabbing [22], healthcare workers could potentially be exposed to viral transmission directly through mucus or aerosolized particles when performing these procedures. Physicians can achieve diagnostic accuracy and prescriptive rationale for point-of-care detection using RIDT through nasopharyngeal swabs in primary care. The present findings show that nasopharyngeal swabbing via the nose was the most common swabbing technique in both seasons.

In the present study, the frequency of vaccinated patients among those with fever was higher during the H1N1-dominant season than the H3N2-dominant season. However, this is not surprising as the efficacy of influenza vaccination has been reported to range from 30 to 80% depending on the circulating virus and vaccination coverage in a given season [23,24,25].

Administering antiviral agents could minimize symptom duration by up to 1.5 days, as well as reduce the complication rates in the lower and upper airways, such as pneumonia or otitis media, when initiated within 36 h of symptom onset [18,26]. However, there is a lack of information regarding the clinical efficacy and side effects specific to each subtype. In our study, most patients were prescribed antiviral drugs following RIDT positivity in both seasons. The antiviral drugs utilized in this study encompassed oral oseltamivir and intravenous peramivir. During data collection, distinctions regarding the route of antiviral drug administration were not considered, which could have potentially influenced the timing of fever resolution. Additionally, it should be noted that other medications, such as anti-febrile agents, might also impact the observed patterns of fever resolution. As a result, caution is warranted when interpreting the results given the potential confounding effects of various medications on the outcomes. However, it is worth noting that the resolution of fever after antiviral drug administration exhibited a significantly more rapid response during the A/H3N2-dominant season compared with the A/H1N1-dominant season. This observation led us to consider the timing of fever resolution as a distinctive characteristic specific to each strain. The findings suggest that A/H3N2 displays a higher sensitivity to fever reduction following antiviral drug treatment. However, from a clinical perspective, antiviral agents and/or vaccination should not be disregarded as effective measures for treating influenza A, irrespective of its subtypes or strains. Minor complications, such as vomiting, were infrequent in both seasons.

In terms of comorbidities and complications, no significant differences were found between the seasons. Therefore, low-virulence viruses may lead to similar comorbidities and complications regardless of subtype.

Our study had several limitations. Firstly, only outpatients were included, which means that severe cases involving patients visiting the emergency room or those who were hospitalized might have been inadvertently excluded. Secondly, there is a possibility that patients with false positives or -negatives were enrolled. RIDT positivity did not always indicate a dominant subtype in each season. Thirdly, it is important to acknowledge that the retrospective design of the study and the inclusion of multiple heterogenous institutions could have introduced certain limitations. Specifically, factors such as differences in test kit types, sample collection site, and other potential confounders were not extensively controlled for. These factors might have influenced the outcomes of the current investigation and potentially diminished its clinical significance. Fourthly, due to the nature of our survey, which focused on the time it takes for fever to subside after taking an ‘antiviral agent’ in 12 h intervals, there is a lack of data from the group that did not take antiviral agents. Nonetheless, considering the clinical context that indicates a notably shorter fever resolution time among patients who took antiviral agents compared to those who did not, we will explore this in future studies. Lastly, we focused our analysis on a single prevailing season for each subtype. Given the considerable variability in influenza A virus strains from year to year, further investigations incorporating multiple dominant seasons of A/H3N2 and A/H1N1 are warranted. This will aid in enhancing the applicability of these findings, ensuring that observed distinctions between A/H3N2 and A/H1N1 are not solely attributed to specific seasonal strains. Given these considerations, well-controlled future studies utilizing larger hospital datasets are warranted to corroborate and validate the present findings. Such research efforts will enhance our understanding of the subject matter and complement the conclusions drawn from this study.

## 5. Conclusions

To our knowledge, the present study is the first to clinically compare two low-virulence strains (H3N2 vs. H1N1). The initial symptoms differed according to the virus subtype. Conversely, upper airway comorbidities and systemic complications showed similarities between the two strains. These symptomatic characteristics should be taken into consideration for accurate diagnosis and effective management of patients with influenza, particularly in primary care settings.

## Figures and Tables

**Table 1:** Study participant demographics.

Variables	2016–2017 Season (A/H3N2 Dominance)	2018–2019 Season (A/H1N1 Dominance)	*p*-Value
Total number of ILI	1897	2191	
Number of RIDT positive	1424	1568	
Age, years	17 (10–37)	27 (14–43)	<0.001
Male, *n* (%)	847 (44.7)	945 (43.1)	0.343
Contact history	89 (4.7)	178 (8.1)	<0.001
Swab site			<0.001
Nasopharynx via nose, *n* (%)	1735 (91.5)	1645 (79.1)	
Oropharynx via mouth, *n* (%)	0	104 (5)	
Nasopharynx via both nose, *n* (%)	0	332 (16.0)	
Nasopharynx and pharynx, *n* (%)	162 (8.5)	0	
RIDT results			<0.001
Positive, *n* (%)	1362 (71.8)	1425 (65.0)	
Weak positive, *n* (%)	62 (3.3)	143 (6.5)	
Negative, *n* (%)	473 (24.9)	623 (28.4)	
Vaccination			<0.001
Before 2 weeks, *n* (%)	384 (22.1)	526 (26.2)	
Within 2 weeks, *n* (%)	32 (1.8)	5 (0.3)	
No vaccination, *n* (%)	1320 (76.0)	1475 (73.5)	

The data are expressed as counts (percentages). Continuous variables were analyzed using the Mann–Whitney U test. Categorical variables were analyzed using the chi-squared test or Fisher’s exact test. ILI, influenza-like illness; RIDT, rapid influenza diagnostic test.

**Table 2:** Initial manifestations in the 2016–2017 season and 2018–2019 season.

Symptoms	2016–2017 Season (A/H3N2 Dominance) *n* = 1424	2018–2019 Season (A/H1N1 Dominance) *n* = 1568	*p*-Value
Fever, *n* (%)	1141 (80.1)	1240 (79.1)	0.496
Average BT, °C	38.1 (37.7–38.6)	37.9 (37.4–38.4)	<0.001
BT ≥ 38 °C, *n* (%)	861 (60.5)	740 (47.2)	<0.001
Myalgia, *n* (%)	686 (48.2)	1126 (71.8)	<0.001
Cough, *n* (%)	580 (40.7)	1001 (63.8)	<0.001
Sore throat, *n* (%)	271 (19.0)	634 (40.4)	<0.001
Diarrhea, *n* (%)	8 (0.6)	12 (0.8)	0.654

The data are expressed as counts (percentages) or numbers (range). Continuous variables were analyzed using the Mann–Whitney U test. Categorical variables were analyzed using the chi-squared test or Fisher’s exact test. BT, body temperature.

**Table 3:** Comparison of clinical efficacy and adverse effects regarding antiviral drugs between two seasons.

Antiviral Drugs	2016–2017 Season (A/H3N2 Dominance) *n* = 1424	2018–2019 Season (A/H1N1 Dominance) *n* = 1568	*p*-Value
Prescription, *n* (%)	1171 (82.2)	1275 (81.3)	0.516
Resolution of fever			
Within 24 h, *n* (%)	840/1171 (71.7)	379/1250 (30.3)	<0.001
More than 24 h, *n* (%)	331/1171 (28.3)	871/1250 (69.7)	<0.001
Vomiting, *n* (%)	26/1171 (2.2)	31/1275 (2.4)	0.763

Continuous variables were analyzed using the Mann–Whitney U test. Categorical variables were analyzed using the chi-squared test or Fisher’s exact test.

**Table 4:** Comparison of upper airway comorbidities and systemic complications between two seasons.

Diseases	2016–2017 Season (A/H3N2 Dominance) *n* = 1424	2018–2019 Season (A/H1N1 Dominance) *n* = 1568	*p*-Value
Upper airway comorbidities			
Tonsillitis, *n* (%)	208 (14.6)	227 (14.5)	0.092
Bronchitis, *n* (%)	148 (10.4)	138 (8.8)	0.139
Sinusitis, *n* (%)	93 (6.5)	104 (6.6)	0.911
Laryngitis, *n* (%)	34 (2.4)	68 (4.3)	0.003
Lymphadenitis, *n* (%)	16 (1.1)	6 (0.4)	0.018
Otitis media, *n* (%)	2 (0.1)	0 (0)	0.226
Systemic complication			
Pneumonia, *n* (%)	1 (0.0)	3 (0.2)	0.626
Hospitalization, *n* (%)	1 (0.0)	4 (0.3)	0.377

Continuous variables were analyzed using the Mann–Whitney U test. Categorical variables were analyzed using the chi-squared test or Fisher’s exact test.

## Data Availability

Data available on request from corresponding author.
